# A Memoir on the Treatment of Bubo

**Published:** 1841-10

**Authors:** 


### WESTERN JOURNAL

OF

### MEDICINE AND SURGERY.

OCTOBER, 1841.

ART. I.—*A Memoir on the Treatment of Bubo.* By DR. MARCHAL (DE CALVI.) Translated from the French for the Western Journal, by THOMAS W. COLESCOTT, M. D., of Louisville.

The name of venereal bubo, or more simply and more generally of bubo, is given to acute or chronic inflammation of the lymphatic ganglia, arising from the venereal virus—an inflammation accompanied, when acute, with that of the surrounding cellular tissue.

The word bubo is derived from the Greek *Boubon*, which signifies groin. Among other inconveniences, it has that of making one suppose that the affection which it serves to designate is developed in the groin alone, whereas it may be produced, though rarely indeed, in other parts. However, as a denomination is advantageous when its meaning is well-settled, and as that of bubo leaves nothing to be desired in this respect, it will in all probability be retained in the science to the exclusion of the term adenitis.

The following is a particular form of abscess, something like bubo, to which Hunter has drawn attention: Sometimes in consequence of ulcers or chancres existing on the glans or the prepuce, the lymphatic vessels of the penis inflame and appear under the form of hard cords, which are lost on the root of the organ, on the pubis, or in the inguinal ganglia. Hunter explained this phenomenon by the thickening of the parietes of these vessels, and the effusion of a quantity of coagulable lymph upon their inner surface. These vessels, thus inflamed and thickened, may suppurate in one or more points, and give rise to one or more abscesses. An analagous phenomenon occurs in simple angio-leucitis,* in which abscesses of greater or less size are observed to occur here and there, at those points where the inflamed vessels meet in great number and form a net work.

Hunter was acquainted with some very rare examples of bubo in the hollow of the axilla. He saw cases in which syphilis had been contracted by a sore or cut upon the finger. When this happens, if a bubo is formed, it occurs at the bend of the arm† or at the inner side of the biceps muscle, rather than in the hollow of the axilla. (S. Cooper.)

The groin being almost the exclusive, and in some degree the natural seat of the venereal bubo, it is to inguinal bubo that we shall confine our remarks. In the groin itself, the seat of bubo is subject to variation. Hunter observed one, in a male, situated very far outwards on the thigh; he has likewise noticed them above Poupart's ligament, and in the

---

*Inflammation of the lymphatic vessels: from *aggeion*, vessel, and *leakon*, white, the termination *itis* being added.—*Tr*.

†Cooper intends to say above the epitrochlea, or inner condyle, as there are no ganglia at the bend of the arm. (Our author has mistaken Cooper's meaning; his expression is: "a little above the bend of the arm, by the side of the biceps muscle, etc." See Dictionary of Prac. Surgery, by S. Cooper; art. Venereal Disease.—*Tr*.)

neighborhood of the pubis. It is an interesting remark, that neither the ganglia of the iliac region, nor those existing along the vertebral column, are ever affected with bubo, which is explained by anatomical reasons (vid. infra.)

In the female the seat of bubo, equally variable, is subordinate to that of chancre. This subordination, as indicated by Mr. Samuel Cooper, in a manner which might be more precise, is as follows: if the chancre exist near the meatus urinarius, the nymphæ, the clitoris, the labia, or the mons veneris, the bubo is found near the place where the round ligament passes out of the abdomen, sometimes lower down in the groin: if the chancre is situated lower down towards the perineum, the ganglionic inflammation manifests itself in the angle resulting from the junction of the thigh with the labium. It will be seen presently that a bubo caused by a pre-existing chancre, is a very rare occurrence. What becomes then of the subordination established by the English author?

Dr. Denis has seen buboes developed in the female, though rarely, at a greater or less distance down the thigh, within two or three fingers' breadth of the top of the member, sometimes in the course of the crural artery, at other times farther inwards.

The ganglia of the groin form a superficial and deep-seated group. Of the former, some receive the vessels of the external genital parts, and those which creep beneath the sub-cutaneous fascia of the abdomen; others, most of the superficial lymphatic vessels of the inferior extremity. Such of them as receive lymphatics from the parts of generation are situated in the fold of the groin or in front of the ligament of Fallopius; the others lower down and within. According to Prof. Velpeau, the former alone would be the seat of bubo proceeding from a virus absorbed by the lymphatics of the genitals. "The others do not swell except under the influence of a disease of the abdominal member." This disease may be a venereal ulcer between the toes; the bubo itself is then syphilitic. But does it never happen that the lymphatic vessels of the parts of generation, by some irregularity, go to the inferior ganglia? and might not these then become the seat of bubo, in consequence of a virus absorbed by the vessels just named?

A distinction relative to the seat, for which we are indebted to Desruelles,* is that which divides buboes into superficial or supra-aponeurotic, and deep seated or sub-aponeurotic; it is important, particularly in relation to the prognosis, as a deep-seated bubo, in which the inflammation is confined by the aponeurosis, must give rise to more intense inflammatory symptoms than a superficial one.

This distinction is opposed to the following opinion of Velpeau:† "The deep-seated ganglia, to the number of three, four, or five, surround the femoral artery, are situated between the two lamina of the fascia lata, and communicate with the preceding (the superficial) by means of minute arteries, veins and lymphatic trunks. As these radicles ought apparently to transmit to the deep-seated ganglia the diseases of the others, it is remarkable that buboes should multiply beneath the skin, and remain there for a considerable time, without any being formed in consequence thereof in the crural canal." Notwithstanding this passage, as direct communications exist between the superficial and deep-seated ganglia, by means, too, of lymphatic vessels, it seems to us possible for the syphilitic virus to be carried from the genitals to the superficial ganglia, and from these to the deep-seated. We think, therefore, that the distinction of Desruelles is not impaired by the anatomical reasons that have just been discussed, and that it deserves to be retained.

We admit that the acute bubo consists rather in inflammation of the surrounding cellular tissue than in that of the ganglion itself. The inflammation of the latter is but the starting point and, if I may say so, the first stage of the bubo.

The following sentence from Velpeau expresses our tho'ts upon this subject very well: "There are two orders of patho-

*Traite pratique des maladies veneriennes: Paris, 1836, p. 527 et suiv.

†Anatomie Chirurgicale, 3d. edit., Paris, 1836, t. ii, p. 528.

logical phenomena. Some, primitive, the product of the specific cause, take place in the ganglia themselves; others, secondary, acting as if they depended on any other cause, have their seat in the surrounding cellular tissue."

It does not follow that the ganglion itself may not suppurate. But suppuration of the ganglion, in bubo, constitutes the exception. The general fact is inflammation and suppuration of the cellular tissue. Velpeau teaches that in cases of inflamed absorbent ganglia, the suppuration may take place, 1st. in the ganglion; 2d. in the cellular tissue subjacent to it; 3rd. in the cellular tissue found in front of it; 4th. in the whole surrounding cellular tissue; 5th. and lastly, at once before, behind, and in the ganglion, which presents a kind of canal through which the pus of the anterior part communicates with that of the posterior. The abscess then presents the form of one of those double buttons used to fasten shirts.

What is the mode, or rather what are the modes of production of the bubo?

A first mode admitted by Hunter is that in which the pus of a gonorrhœa or a chancre, applied to a healthy surface, does not produce any local effect, but is directly absorbed. This mode, according to Hunter himself, is extremely rare. We admit perfectly that a bubo may be thus directly formed, without any other venereal symptoms being produced; but we do not think that a bubo can ever be produced by the pus of gonorrhœa, so that we adopt the proposition of Hunter with a reservation.

The most frequent mode, and the one which constitutes the rule, is that in which a chancre has existed prior to the bubo, or that in which a chancre and a bubo are developed at the same time. In this last case, it is very evident there can be no relation of causality between the two. The mode of production is the same as above; only the bubo is accompanied with a chancre.

When the chancre has existed previous to the bubo, a question is presented: Has the chancre given rise to the bubo, or were the two symptoms, as in the preceding case, produced at the same time, although one appeared before the other, a circumstance readily conceived, seeing that, to produce bubo, the virus had to pass through a certain extent of lymphatic vessels, whilst for the chancre no such thing was necessary? We think both circumstances may occur. However, if it is considered that, very frequently, the chancre which precedes a bubo is scarcely perceptible, that, consequently, it furnishes but a very small quantity of pus for absorption, supposing it to furnish any, it will be admitted that when first a chancre and then a bubo are formed after impure coition, they are most frequently a simultaneous effect, and not the effect of one another. It may be seen, from this, how rare consecutive buboes would be, that is to say, those produced by a chancre already existing in the same individual. To recapitulate, it seems to us the following propositions may be established relative to the production of bubo:

1. A bubo may be communicated directly from the male to the female and from the female to the male by the pus of a chancre, without any other symptom.

2. A bubo may be produced directly and a chancre be produced at the same time, so that they are manifested simultaneously, independently of each other.

3. A bubo may be produced directly, and a chancre be developed at the same time, independently of each other, in such a manner, however, that the chancre is manifested before the bubo.

4. A chancre may give rise to a bubo in the same individual.

Hunter, Assalini, and several other surgeons, have observed that mercurial frictions sometimes give rise to ganglionic engorgements in the inguinal region.

It is generally easy to distinguish the swelling produced by an inflammation of the ganglia of the groin from any other tumor of that region. Let us not forget, though, that this inguinal region, so interesting and so complicated in a surgical point of view, has been the seat of very serious errors. Cullerier, supposing that he was about to open a bubo, plunged a bistoury into an aneurismal tumor. This is a case that Prof. Marjolin never fails to relate in giving the history of abscesses. The surgeon recovered from his surprise, but nothing was prepared for so dreadful an occurrence. The wound was plugged with charpie thrust into the cavity of the tumor, and a compressive bandage applied. The hemorrhage was arrested, and the surgeon was happy enough in saving his patient, who eventually got well.

In relation to herniæ, two things may occur: the bubo may be mistaken for a hernia, or a hernia be mistaken for a bubo. The first happened to Sabatier. The case was that of an officer who, after a violent exertion, had observed an indolent tumor appearing in the inguinal region, without any change of color in the skin; he had, besides, no suspicion of the venereal affection. Sabatier directed a truss to be applied; the symptoms became more decided, and the mistake was discovered. Here every thing had concurred to obscure the diagnosis. Should not the circumstance of the exertion have seemed decisive? We have said that in other cases, and more frequently, a hernia is mistaken for a bubo. The observation related by Louis, in his memoir on herniæ with gangrene, is well known. Dr. Burdin, the younger, (see the memoir of Dr. Mondiere, in the *Archives*, 2nd series, vol. iv,) has related the case of a man who carried in the groin a tumor that was unattended by any change of color in the skin, and was mistaken for a bubo by one of the most celebrated surgeons of the capital. Other physicians were not of this opinion ; the patient, on his part, affirmed that he had not been exposed to the venereal infection. The tumor was opened; a worm and some fecal matter passed out. A case nearly similar to this is likewise reported by Mondiere, who says he had it from Dezeimeris. It will be observed that the error is not equally serious, to mistake a bubo for a hernia, or a hernia for a bubo. In the former case, the patient may be made to suffer by the application of a truss; but this is almost the only mischievous occurrence that can befall him. In the other, the tumor may be cut into, and a fold of intestine opened.

The possibility of such errors, and, above all, the fact that surgeons of the first merit have committed them, should put one on his guard. It is unnecessary ever to exaggerate the difficulties of diagnosis; for a person never should act lightly and without having reflected on the chances of error.

If, in most cases, it is easy for the surgeon to recognize that he has to deal with an engorgement of the inguinal ganglia, still it may be difficult to learn the nature of that engorgement. If a chancre exists, and there is at the same time inguinal pain, the question is not doubtful. If there is gonorrhœa, it may still be said that the patient was exposed to venereal contagion; the only point then will be to know if the bubo is of the same nature as when a chancre exists or has existed, a question which will be examined presently. If there is neither chancre nor gonorrhœa, and if the penis bear no cicatrix that would refer to the former existence of a chancre, it may be very difficult to come to a decision. We have admitted with Hunter, as the reader has already seen, the possibility of primitive buboes, but we assert that real difficulties attend the surgeon in their diagnosis. Often he will have a suspicion equivalent in his own mind to a certainty, and yet he dare not, he must not express it, for, despite all appearances, the charge he would bring against the patient might be unjust. Let us speak only of what takes place in a body of soldiery. When a man has been sent to the hospital on account of a venereal affection, at the time of his discharge, he is punished by being made to stand guard fifteen days;* sometimes indeed the punishment is more rigorous. This, it may be said in passing, is a statutory provision questionable in its justice, and liable to be impugned as to its results. It would seem that a man is sufficiently punished for his inability to resist the impulses of youth, by the sufferings he has experienced and by a long sojourn in a hospital. On the other hand, the measure in question remedies nothing; the officers

*The original runs thus: "*il est puni de quinze jours de consigne:*" it is not without some misgivings that I have ventured to translate it as above.—*Tr.*

who enforce it, do so merely in obedience to received orders, persuaded that the punishment inflicted is completely useless, useless for the individual, who will relapse as soon as he shall have the desire, and useless, by a still stronger reason, as an example. In short, not only is this measure ineffectual, it is likewise dangerous, because, to avoid being punished, the soldiers sometimes succeed, notwithstanding inspection, in concealing their disease, and treat their own cases, or abandon themselves to the rapacious ignorance of charlatans. Be this as it may, let us suppose that, in a soldier, who will be punished should his malady be pronounced venereal, an inflammation of the inguinal glands is developed, without there existing either chancre or gonorrhœa; let us suppose furthermore, of course, that the patient declares he has not been exposed to the contagion; how can it be affirmed that a bubo exists?

If it were true that bubo had no other seat than the ganglia in the fold of the groin and in front of Poupart's ligament, and that these could inflame under the influence of the syphilitic virus only, there would no longer be any question. But, on the one hand, we have seen that bubo may be developed lower down; on the other hand, nothing proves that the inguinal ganglia may not inflame from any other cause than the venereal virus, for example, in consequence of a prick of the foot; so that the difficulty remains the same. Happily it is rarely presented; the venereal bubo, without previous or concomitant chancre, whereby it may be known, being itself very rare.

The bubo caused by the virulent pus of a chancre, no matter by what mode in other respects it has been produced, differs essentially from that which may result from gonorrhœa. In the former case, there has been infection; it is a virus absorbed and carried into the ganglia, that has produced the disease: in the second, the ganglia have been irritated and inflamed through sympathy; it is irritation of the urethral canal that has produced the ganglionic inflammation in the groin, just as it frequently happens that a puncture of the finger is followed by the formation of a phlegmon in the axilla, beginning in the axillary ganglia. Both diseases are venereal, since, whether there was chancre or gonorrhœa, the cause was venereal; but one is venereal from its cause as well as from its nature, the other from its cause alone, and its nature is exclusively inflammatory.

Passing by the symptoms of bubo, which we suppose are known, we come to the treatment, in which we shall have in view exclusively the bubo essentially venereal, the bubo properly so called, that is to say, the one which results from the virulent pus of a chancre. It is necessary to bear in mind that there are always two elements in this bubo, the virulent and the inflammatory, which are the product of one another. It is useless to call in question one of these merely to admit the other. To see in a bubo none but the inflammatory element, is to see but half the disease; it is to recognize the effect to the exclusion of the cause. It is not more possible to deny the venereal virus in its results, than the spermatic liquid in its results. Syphilitic affections are propagated by means of their virus, just as the animal races are by means of the seminal liquor. It is in both cases the same power in a reproduction faithful to its primitive types.

Bubo is acute or chronic, openly inflammatory or indolent. The treatment differs in these two cases.

*Treatment of the inflammatory or acute bubo.*—The object of this treatment is to disperse, to break up the inflammation, or, the termination by suppuration being unavoidable, to evacuate the tumor, and induce cicatrization of the wound. It is therefore abortive or curative.

*Abortive treatment.*—This should always be tried when the patient presents himself at a stage in which resolution is still possible. There is an idea among the vulgar that if a bubo be prevented from suppurating, the virus remains in the economy. This idea is not without foundation. When the surgeon, neglecting the generative element, essential to the bubo, merely combats the inflammatory symptoms, it is very possible that the virus will remain. It need not be said that these are old doctrines unworthy of being revived. To determine inflammation, the venereal virus, like any other irritative agent, requires blood; if leeches are applied on the point where it has been carried, and if, by this means, aided by some others, it is deprived of the necessary sanguineous element, it will most assuredly remain inactive at the point indicated; but there is nothing to show it may not be absorbed, and exert its disastrous influence elsewhere. It is our opinion, therefore, that, whatever be the means locally employed, unless the mercurial ointment is applied by frictions, which will be considered below, recourse must be had at the same time to general remedies, capable of acting upon the secretions or in a specific manner, and thus of expelling or neutralizing the virulent principle. We do not believe that the eighth of a grain of the deuto-chloride or proto-iodide of mercury, for a fortnight, and the use of sarsaparilla for a month, can, in any case, produce bad results. On the contrary, we have seen the most advantageous alterations effected in the general nutrition under the influence of these remedies. Frequently, in patients submitted to their use, not only do the inflammatory symptoms diminish, but likewise the appetite augments, and very soon the plumpness becomes decided.

In a garrison where we were detached with a battalion, we treated, at the infirmary of the regiment, a number of buboes in the formative stage; and the following is the method pursued by us: deep cauterization of the chancre with the acid nitrate of mercury, repeated according to necessity; application of twenty leeches to the bubo; from the beginning, a pill containing one-eighth of a grain of the proto-iodide of mercury every evening, and a pot of the decoction of sarsaparilla every day. In other respects, the antiphlogistic regimen as long as the inflammatory symptoms lasted, then the ordinary regimen. This mode of treatment has succeeded perfectly in our hands.

If the inflammatory symptoms are not sufficiently modified by the first application of leeches, no fears need be entertained of repeating it one or more times, since if the local inflammation does not succeed in resolving the bubo, it still serves to limit its extent and diminish inflammation.

The object in cauterizing is not merely to prevent the secretion of virulent matter by the chancre, but likewise to induce its prompt cicatrization. If, however, the chancre be pretty large and inflamed, cauterization must be laid aside, although it is more successful than one would suppose even in such as are inflamed.

After leeching, some surgeons advise the application of ice. In a case where it was of great importance that a bubo and a chancre of the prepuce should be got rid of as quickly as possible, we directed twenty leeches to be applied, and followed during two days by ice; the cure of the bubo was complete at the end of that time; the chancre, which was cauterized twice, did not heal for eight days. No conclusion should be drawn from this case, which is certainly exceptional. Dr. Denis has in several instances seen the symptoms exasperated under the influence of ice, and has renounced the remedy.

Hunter, in order to bring about the resolution of the bubo, employed mercurial frictions upon it. He thought that resolution depended on the amount of mercury that might be absorbed, and admitted that certain buboes were too little developed to take up a sufficient quantity. In this case, he advised the frictions to be extended to the thighs. The mercurial inunctions, according to him, cured at once the ganglionic inflammation and the general infection. Delpech was equally partial to the use of this remedy, which, however, is not now employed in France, at least not as a means of resolving the acute bubo, since, if it is capable of modifying the virulent element, it acts less promptly and much less surely against the inflammatory element, than local blood-lettings, in conjunction with emollient applications.

Simple vesication, as a discutient remedy, does not seem to us to be any more productive of advantageous results. We have seen a blister, applied to an inguinal bubo in an officer, bring on suppuration, though the tumor appeared to have a manifest tendency to resolution.

The only means of rendering the blister pretty certainly discutient or resolvent would be, perhaps, to make it of enormous size, like those long ago successfully employed by Prof. Velpeau at La Charité, and which he significantly terms *monster-blisters* (*'vesicatoires-monstres.'*) Otherwise, we do not hesitate to affirm that vesication, instead of being resolutive is essentially maturative. In no case ought the blister to suppurate. Indeed, if it is conceivable that an acute irritation, sudden and extended over the skin, could violently remove the inflammation of the sub-jacent ganglia, this is not understood of a deep and slow irritation like that of a suppurating blister, which is far more capable of producing an inflammation of the neighboring ganglia than of dispersing it.

Another discutient means has been indicated by surgeon-major Malapert and adopted by Dr. Renaud of Toulouse. It consists in the application of a blister the size of a three-franc piece, and dressing the denuded surface with a solution of the deuto-chloride of mercury. An eschar is formed the thickness of which, owing to the presence of a certain quantity of lymph, is greater than that of the portion of skin destroyed. Ricord has given to this method the name of *mediate cauterization.* It is extremely painful. There are patients who, with the utmost resignation, cannot keep the pledget saturated with the sublimate. Besides, the employment of this method leaves a very extensive, indelible cicatrix, an unwelcome result which the surgeon ought to use all his care to avoid. Lastly, it is often ineffective. In a number of cases in which it was tried at the hospital of Gros-Caillou, by Denis, and at the military Hospital, by Desruelles, it did not prevent the bubo from suppurating. Therefore, although it has obtained the sanction of Ricord, and originated with one of our most distinguished colleagues, we do not hesitate to give our verdict against a method which has the double inconvenience of being uncertain and very painful.

Many practitioners make use of compression after the inflammatory symptoms have been sufficiently modified. Dr. Denis frequently employs this means after one or two applications of leeches, and derives good effects from it.

Hunter, according to Mr. Samuel Cooper, (Dict. of Practic. Surgery, art. Syphilis,) was favorable to the use of emetics in the treatment of bubo, and accorded to them the power of resolving the ganglionic inflammation, even after pus was distinctly formed. We merely refer to this as the opinion of a great surgeon. In France, the tartarized antimony has never been applied to the treatment of bubo.

A last discutient remedy has been recently advised, and consists in multiplied punctures. The punctures with a lancet act, says Dr. Aubry,* like true local blood-lettings, by relieving the engorgement of the parts; accordingly there should be no fear of making them deep; experience indeed has demonstrated that it is important for the point to penetrate the ganglia, and divide the fibrous envelope. This means, since the notice of Aubry, has been tried by several surgeons, among others by Barthélemy, who has charge of the venereal wards in the military hospital of Picpus, and it seems to have been productive of good effects. The surgeon just mentioned is at this moment making trials upon a large scale. We cannot help thinking, however, leaving our opinion to be corrected by future facts, that punctures, as a means of procuring resolution, must be a less certain mode of treatment than that by antiphlogistics and specifics, to which, until more ample information is obtained, we give the preference.

*Curative Treatment.*—When the patient has been tardy in presenting himself to the surgeon, or when the means proper for arresting the inflammatory action have failed, an attempt must be made to moderate the inflammation and limit the suppuration.

It seldom becomes necessary to bleed individuals affected with bubo. Leeches, on the contrary, may, and often, must be employed, they having the power, more than any other remedy, of restricting the phlogistic effects of the virus. Cataplasms of ground flax-seed, or, if these irritate the skin, of bread and milk, should be applied lukewarm to the tumor. Rest in the horizontal posture, and flexion of the limb should

*Gazette Medicale. August 15, 1840.

be prescribed, especially if the bubo be deep-seated. The patient should use the tepid bath: local bathings, says Ricord, and this is likewise the opinion of Dr. Denis, are of very little service. In addition, mild purgatives ought to be used, according to necessity, so that the flow of venous blood in the parts around the pelvis may not be obstructed by the accumulation of fæcal matter. The patient should be put upon a mild diet. When nothing opposes, he should take daily a quart of the decoction of sarsaparilla.

Such, briefly, are the means to be employed until fluctuation indicates that pus has been formed to which it is time to give vent. Authors have established important rules respecting the mode of ascertaining the fluctuation.

In some cases, fluctuation exists at the centre only of the inflamed mass, and then it forms, according to the expression of Dr. Ricord, a sort of *artesian well.* A person may be deceived, though hardly if he has any experience, by the softness of the ganglionic tissue, and believe pus to be present when there is none. The quantity of matter that a bubo encloses is a frequent source of deception, as no one would imagine it to contain as much as it does, particularly when, being sub-aponeurotic, it spreads under the aponeurosis, and gains in breadth and depth what it lacks in heighth externally.

Let us suppose fluctuation to be well established. Hunter was for waiting until the skin became very thin, a precept that we cannot adopt; in fact, to wait until the skin is thinned, is to condemn it to inevitable destruction by ulceration, or by the means of art.

The moment having arrived to open the bubo, how is the operation to be performed? This leads us to speak of the method by punctures, pursued at the military hospital of Gros-Caillou, in the wards of Dr. Denis, the surgeon-major, one of those enlightened and conscientious practitioners whom it is necessary to make known, because they are too modest to reveal themselves. The note of Dr. Aubry apprises us that Cullerier has been employing multiplied punctures in the treatment of bubo for a long time at the hospital *du Midi;* it is certain that this means has been used by Denis for at least fifteen years. The question of priority remains, therefore, in suspense.

In the beginning, Dr. Denis employs the antiphlogistic treatment, according to the intensity of the inflammatory symptoms. He has observed that mercury, during the period of irritation, had a great tendency to act upon the gums, and he abstains from its administration at that period. We ought to remark that we do not share the apprehensions of Dr. Denis in this matter, since, if the metal is employed with moderation, it is not difficult to arrest the untoward effects it may produce on the mouth, and besides, when it has been thus wisely administered, it very seldom occasions them.

The necessity of caustic generally betrays the surgeon, as it depends solely upon the extreme tenuity of the skin, a portion of which has to be removed, and as this tenuity itself results from the delay in opening the abscess. The application of caustic creates a considerable sore, which is slow to heal, and, after recovery, leaves a much more striking cicatrix than when a cutting instrument has been employed. The latter, therefore, unless from timidity on the part of the patient or extreme thinness of the skin, should be preferred to the caustic.

Now, how should the bistoury be used in opening a bubo? An aperture of greater or less extent is generally made along the bend of the groin, or in the vertical direction, as Velpeau often makes it, and in our opinion more rationally. When the bubo is of small size, it is recommended to practice a simple puncture. But the cases in which this is possible are rare. Frequently when this has been done, the pus has been known to stagnate and detach the adjacent parts. The most frequent practice consists then in making an incision of greater or less length, sometimes very extensive; now experience proves that incisions of this kind, although far preferable to the caustic, require a long time to cicatrize, often render excision of the edges of the wound necessary, and leave a very marked cicatrix, all of which inconveniences the process employed by Dr. Denis is designed to avoid. This process is as follows:

Instead of a single very extensive incision, he makes several punctures, the number of which varies from two to three or four, according to the extent of the tumor, and what is important to notice, corresponding to the extremities of the bubo, so as to give exit to the pus at the moment it is about to spread among the adjacent parts and detach them. If the bubo does not exceed the ordinary size, he is satisfied with two punctures at the extremities of its greatest diameter; if it is very considerable, he makes one or two others at the extremities of the opposite diameter. During the first few days, some bits of charpie are introduced into the small openings, to keep them from closing. In those very rare cases in which a single puncture will suffice, Dr. Denis acts in the ordinary manner. It is proper to make the punctures perpendicular or oblique in reference to the fold of the groin, as is done by Cullerier.

The punctures being disposed at the extremities of the bubo, a slight pressure at the centre suffices to evacuate it, and the skin is not involved at the point where it is thinnest, and where there is most need of preserving the vessels; from this two-fold circumstance results an easy detergence and the prompt re-attachment of the fundus of the abscess to the teguments which have retained their vitality, so that, finally, complete cicatrization ordinarily occurs from eight to ten days after the operation. A striking advantage, the greatest perhaps attached to the multiplied punctures, is that the cicatrices are scarcely discernible, often imperceptible. Indeed, it was in a great measure the desire to avoid, in females particularly, large and deep scars, lasting stigmas of shame, that led Dr. Denis to conceive this process.

The very simple method of operating just indicated might be very well applied, in a great many cases, to the opening of ordinary phlegmons, and we do not doubt that it will come into very general use.

A method that resembles the preceding as to its results, though it differs remarkably as to the means, is precisely that of surgeon-major Malapert, which has already been considered. When the blister is applied, and afterwards the solution of corrosive sublimate, in a case where suppuration takes place, small eschars are observed at the surface of the bubo that soon fall off, and leave openings through which the pus makes its escape. This mode of treatment has been particularly employed by Renaud, from whom it was copied by Vidal (de Cassis), who has frequently used it with success in the venereal hospital. Considered in itself, this method is advantageous; compared with the multiple punctures, it is much inferior. We have already said how painful it is.

Vidal, who has also employed the method by puncturing, has several times used the scarificator in practicing it.

Dr. Denis does not prescribe mercury to all his patients affected with bubo. In the administration of this article, he is guided by the aspect of the sore, its tendency to cicatrization, and the general health of the individual. Certainly there are buboes which heal perfectly without mercury: Hunter, who was a great partizan of the specific metal, admits this; we think, however, that there is more security in employing it in every case. We need not add, that when Dr. Denis makes use of the deuto-chloride of mercury, or the proto-iodide, which he prefers, it is always with the prudent reserve of a practitioner who is thoroughly acquainted with the medicine that he prescribes. The dose varies from an eighth to a quarter of a grain daily, and seldom amounts to half a grain. In females, it is most frequently limited to one-eighth.

The fault of the doctrine which proscribes this specific is in speaking as if the world had not profited by the experience of fifty years. At this day, no person abuses mercury. Why should any one be so obstinate as not to perceive it, and what is this strange mania of making war, like some famous knight-errant, upon excesses that have long been imaginary?

*Treatment of the chronic or indolent bubo.*—We shall be brief in what remains to be said, as the task we proposed in the outset is fulfilled. The chronic or indolent bubo is primitive or consecutive. It is in this form that the repeated application of blisters is particularly efficacious. They act by inducing suppuration or resolution of the ganglia. Mercurial or ammoniacal frictions, emollients, douches, long walks, are so many means suitable to resolve indurated ganglia, or to inflame them and bring on suppuration. Compression, combined with vesicatories or irritating frictions, constitutes a mode of treatment by means of which we have seen Dr. Denis obtain the resolution of indolent buboes of considerable size. If the means thus rapidly pointed out do not produce the desired effect, inflammation may be directly excited by an issue of caustic potassa. The ganglia may also be excised, which is preferable, in imitation of Desruelles, and subsequently cauterized, or leeches may be applied after the excision, which ought to be tried first. Here again punctures are attended with beneficial results. "It is," says Aubry, "in cases of indurated chronic buboes particularly, that Cullerier advises the ganglion to be pierced deeply; this charge is important, and its omission may compromise the success of the method." It is sometimes necessary, in cases of this kind, to repeat the punctures. In this matter, we should be guided by the progress of the disease. Aubry cites three examples of very large buboes cured at the end of a month. It is probable that, from analogy, the multiple punctures will shortly be applied to scrofulous tumors.

Buboes are susceptible of several complications. A phlegmonous erysipelas may occur after the application of leeches. The surgeon now and then meets with individuals of a scrofulous constitution, in whom cicatrization proceeds with extreme slowness. Some buboes are accompanied with an ulceration truly phagedenic, a terrible symptom with which we saw a young soldier die at Val-de-Grâce, although he had been subjected to simple treatment. Finally, intercurrent diseases exert a great influence on the progress of the bubo. But these various complications do not enter into the plan we have traced out for ourselves.

